# Consumption of Zinc Biofortified Rice Diet Improved Mineral Availability: An Approach to Address Micronutrient Malnutrition

**DOI:** 10.3390/foods15142567

**Published:** 2026-07-22

**Authors:** Natesan Ramachandran, Marappan Gopi, Duraisamy Rajendran, Dintaran Pal, Balakrishnan Binsila, Madhusoodanan N. Unnikrishnan, Chirravuri N. Neeraja, Gundallahalli B. Manjunatha Reddy, Arindam Dhali, Sudhir Chandra Roy, Ashish Mishra, Kaliappan Bharathi, Jeeva Mutthu Kumar, Artabandhu Sahoo, Raghavendra Bhatta

**Affiliations:** 1 ICAR—National Institute of Animal Nutrition and Physiology, Bengaluru 560030, India; getgopi72@gmail.com (M.G.); dtpal@yahoo.co.in (D.P.); drbinsila@gmail.com (B.B.); unnikrishnan1420@gmail.com (M.N.U.); scroy67@gmail.com (S.C.R.); ashishvet1@gmail.com (A.M.); bharathinjagan@yahoo.com (K.B.); jmutthukumar.2@gmail.com (J.M.K.); sahooarta1@gmail.com (A.S.); 2 ICAR—Indian Institute of Rice Research, Hyderabad 500030, India; cnneeraja@gmail.com; 3 ICAR—National Institute of Veterinary Epidemiology and Disease Informatics, Bengaluru 560119, India; gbmpatho@gmail.com; 4 ICAR—National Dairy Research Institute, Southern Regional Station, Bengaluru 560030, India; dhali72@gmail.com; 5 Indian Council of Agricultural Research, New Delhi 110012, India

**Keywords:** Zn-biofortified rice, feed pellets, biologically available zinc, retention, tissue distribution, rats

## Abstract

Zinc (Zn), a micronutrient, plays vital roles in various biological functions and its inadequate intake results in various clinical manifestations. Biofortification of staple crops through breeding approaches has been attempted to ensure a higher dietary intake of Zn. The present study assessed the bioavailability of zinc from biofortified rice in Wistar rats. Two rice-based feed pellets were prepared: conventional/control (CR—MTU1010) and Zn-biofortified rice (ZnBR—DRR Dhan 48). Rats were fed their respective diets for 45 days to assess the Zn intake, retention, distribution and excretion. Daily feed intake and weekly body weight were recorded, and a digestion trial was conducted during last five days of the feeding period. Mean body weights at the start and end of experiment were comparable (*p* > 0.05) (246 and 240 g) with similar feed consumption. Zn absorption from ZnBR was 45.12%, compared to 26.65% from CR. The biologically available zinc from ZnBR was 2.3 times higher than that from CR. Zn content in the major storage tissues was not altered (*p* > 0.05) due to consumption of ZnBR. The study revealed that biological availability of zinc from ZnBR was higher than that of CR, which could be useful in managing Zn-related malnutrition.

## 1. Introduction

Malnutrition due to insufficient micronutrient intake ranks second among the seventeen Sustainable Development Goals of the United Nations (UN-SDG2), which aim to eliminate all forms of hunger by 2030. Micronutrient deficiency affects nearly one-third of the global population, and severely affects the economic growth of developing countries [1]. Deficiencies in micronutrients, especially zinc (Zn) and iron (Fe), are one of the major challenges associated with human health in the 21st century [2]. Among the micronutrients, the importance of microminerals is often overlooked, but they play key roles in various biological pathways. Studies have indicated that about 30% of the global population consumes a Zn-deficient diet and is at risk of malnutrition, particularly children [3,4]. The study further indicated that in countries with prevalent Zn deficiency, the basal diet is mainly based on cereals that are grown in Zn-deficient soils. A recent national-level survey in India indicates that the soil Zn content is less than 0.6 ppm; this deficiency ranges from 2 to 67% across different states, which causes an average deficiency of approximately 39% in the crops produced [5]. The soil Zn deficiency has increased to 63% from 42% over the past five decades [6].

Zinc is an essential micronutrient, and it is crucial for various biological functions, including serving as an enzyme co-factor, preserving the biomolecule structure, maintaining membrane integrity, eliminating free radicals, and facilitating gene expression and translation to proteins [7,8,9]. Zn deficiency affects all segments of the population, including infants, children, adolescents, and pregnant women. The clinical symptoms include stunted growth, compromised immune response, weakness, anaemia, skin lesions, and altered endocrine, nervous and reproductive functions [10]. The majority of the Indian population meets their calorie intake through rice (73%) and wheat (62%) [9]. Cereals have the least Zn content, compared to pulses and protein sources of animal origin [11]. In addition to their low Zn content, they also contain phytate, which directly interferes with the availability of bivalent cations (Ca, Zn, and Fe) to the body by forming insoluble mineral–phytate complexes. The processing of cereals, especially the polishing of rice, further increases mineral loss. The cereals grown in Zn-deficient soils were found to have less Zn content compared to those grown in normal soils [4]. Considering these factors, increasing dietary Zn intake through fortified cereals could be an effective strategy to tackle Zn deficiency related to physiological and health complications in large populations.

Increasing the content of micronutrients in food crops through agricultural technologies is advocated as a pivotal means to reduce micronutrient malnutrition [12]. Biofortification is a feasible and cost-effective way to deliver limited amounts of nutrients to populations [13], and is a sustainable alternative that can be highly beneficial for people with limited access to other dietary sources [14]. Biofortification of staple crops with specific nutrients, such as Zn, is expected to reduce the dietary Zn inadequacy without increasing food intake. Apart from mineral enrichment, biofortification is also reported to reduce the phytate content in grains, resulting in increased Zn bioavailability [15]. Biofortification is expected to increase Zn content and its bioavailability, allowing similar levels of consumption of food to meet the requirements for children and pregnant women.

The Indian Council of Agricultural Research has developed biofortified rice varieties with higher Zn content. The variety developed considered here (DRR 48) contains 24 ppm Zn, compared to the conventional non-fortified variety with 12 ppm, and shows a higher productivity of 52 quintals per hectare [16]. Considering its Zn content and yield, the biofortified rice variety DRR 48 has long-term potential for introduction into the public distribution system to alleviate Zn malnutrition. However, before use for human consumption, the variety needs to be evaluated for its mineral bioavailability. Moreover, it is not always the higher content that matters, but rather the bioavailability. Studies carried out with Zn-biofortified rice indicated higher Zn content (~56% increase) in rice, as well as mineral uptake by the differentiating CaCo-2 cells, under in vitro systems [17]. Further studies on Zn-deficient rats have revealed that Zn-biofortified (ZnBR) feed significantly reversed suppressed growth in Wistar rats [18]. The Zn homeostasis in both humans and animals is maintained through intestinal absorption and endogenous faecal excretion in relation to dietary Zn intake. Similarly, a secondary homeostatic mechanism has been reported, involving alteration in urinary Zn loss relative to plasma zinc turnover and transfer across major storage tissues such as bone and skeletal muscles [19]. Under the conditions in India, malnutrition is prevalent, instead of simple deficiency, and studies on mineral availability from Zn-biofortified rice as a staple diet under an in vivo system are lacking. Therefore, the present study was carried out to assess the impact of complete replacement of conventional rice with Zn-biofortified rice on the bioavailability of Zn, the distribution patterns of major bivalent cations, and antioxidant activity, in addition to potential histological changes in the vital organs of Wistar rats.

## 2. Materials and Methods

### 2.1. Ethical Approval

All the experimental protocols and procedures performed in the study were as per the approved guidelines of the Institute Animal Ethics Committee (NIANP/IAEC/1/2022/5).

### 2.2. Experimental Animals, Conditions and Feed Ingredients

**Figure 1:**
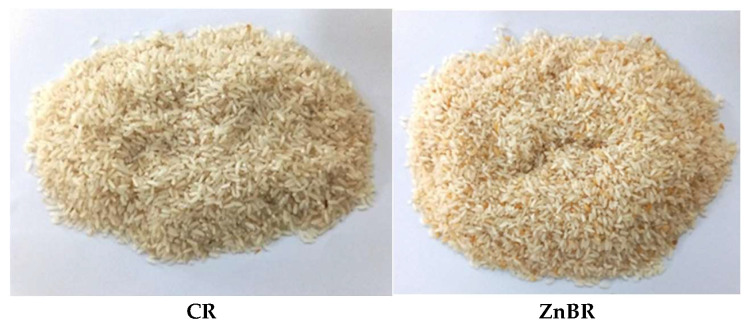
Polished rice varieties. CR: Conventional rice; ZnBR: Zinc-biofortified rice.

The experimental animals used in the study, Wistar rats, were produced in-house at the Central Laboratory Animal Research facility. A parent stock of same age group was bred to obtain pups from a single harvest cycle. The pups were allowed to suckle their dams and were weaned on day 21. A total of 40 weaned rats (20 males and 20 females) having similar body weight (42 ± 2 g) were used for the study (42.64 ± 0.93 g; 43.36 ± 1.20 g, *p* = 0.256). The rats were randomly assigned to both groups, with 20 animals per group (10 males and 10 females), and housed in five replicate polypropylene cages, each containing two rats of the same sex. The replicate cages of each group were placed at different locations on the holding racks to avoid spatial micro-climatic variations. Throughout the study period, the rats were maintained in a BSL-2 facility under the micro-climatic conditions of 22 ± 2 °C; 50 ± 5% relative humidity; 10–15 cycles of air exchange per hour; a positive pressure difference of ~10 Pa; and a 12 h light:dark cycle. The rats had ad libitum access to feed and water, and were regularly monitored for their activities. Both the conventional/control (CR; variety: MTU1010) and zinc-biofortified (ZnBR; variety: DRR Dhan 48) rice varieties (polished) were procured from the ICAR-Indian Institute of Rice Research, Hyderabad (Figure 1). The ZnBR was developed through conventional cross-breeding methods. The other feed ingredients, such as soybean meal, vegetable oils, and vitamins and Zn-free mineral mixture supplements, were procured from local the market.

### 2.3. Diet Formulation and Experimental Diet Preparation

Two rice-based rat diets were formulated to meet nutrient requirements according to ICAR recommendations [20]. The diets consisted of 24% soybean meal, 10% vegetable oil, 0.5% vitamins, 3.5% minerals and 60% rice, either conventional non-fortified (CR) or the Zn-biofortified (ZnBR) variety. Both diets comprised similar calorie (*iso-calorific)* and protein (*iso-nitrogenous)* contents. The Zn-free mineral mixture was formulated, and all the mineral supplements were ground and prepared by mixing in a cone blender. The composition of the Zn-free mineral mixture used in the experiment (g/kg) was as follows: dicalcium phosphate (Dibasic)—500 g; potassium citrate monohydrate—220 g; sodium chloride—74 g; potassium sulphate—52 g; magnesium oxide—24 g; ferric citrate—6 g; manganous carbonate—3.50 g; chromium potassium sulphate—0.55 g; potassium iodate—0.01 g; sodium selenite—0.01 g; and sucrose—119 g. The raw materials, rice and soybean meal were finely ground and sieved. All the ground ingredients, oil, mineral mixture and vitamins were mixed, and a dough was prepared with warm water (60 °C). The dough was then pressed through a locally fabricated 18 mm stainless steel die to obtain pellets of 4 cm length by manual operation. Uniform-sized pellets were prepared at fortnightly intervals, allowed to dry at low temperature, and then stored in an airtight container until feeding. The rats were offered pre-weighed feed pellets once daily in the morning.

### 2.4. Experimental Trial, Sampling and Storage

Rats were fed experimental diets for 7 days during the initial adaptation period, and the experiment was carried out for 45 days. Feed consumption was recorded daily, and body weight was measured weekly. A digestion trial was conducted for five days at the end of the feeding trial. The rats were sacrificed, by using xylazine and ketamine anaesthesia, to collect blood, vital organs, muscles and bones. Blood was collected using a heparinzed needle and syringe, transferred to vacutainer tubes, and kept in an ice box until centrifugation at 10,000 rpm for 10 min using a refrigerated centrifuge (5430 R; Eppendorf, Hamburg, Germany). Plasma samples were aspirated and stored at −80 °C until further analysis. The bones (tibias), skeletal muscles (thigh), liver, kidney, intestine (jejunum), and spleen were separated, washed in normal saline buffer, and stored at −80 °C until further analysis. The organs for histological assessment were stored in 10% formal saline solution at room temperature.

### 2.5. Chemical and Mineral Composition of Rice, Diet and Tissues

The chemicals used in the experiments were procured from Sigma-Aldrich Co., St. Louis, MO, USA, unless otherwise mentioned. The ground samples of feed offered, feed residues left, and faeces were analysed for the proximate principles according to the standard procedures of the Association of Official Analytical Chemists [20]. Based on the proximate principles, the contents of dry matter (DM), organic matter (OM), crude protein (CP), crude fibre (CF), and ether extract (EE), as well as the nitrogen (N) balance, were estimated. The contents of Zn, iron (Fe) and calcium (Ca) in the rice, experimental diets, plasma, bone, intestine, kidney, and liver were estimated using the ICP-OES (Optima 8000; Perkin Elmer, CT, USA) against known standards. Briefly, the rice and feed samples were ground and ashed using a muffle furnace. The mineral extract was prepared using 3N hydrochloric acid. Similarly, the tissue samples (muscle, intestine and liver) were processed using the dry ashing method [21]. The bone samples were thawed, defatted for 30 h using acetone, dried in a hot air oven at 60 °C for 12 h, and weighed before ashing.

### 2.6. Antioxidant Enzyme Activity

The plasma catalase (CAT) activity was determined spectrophotometrically through neutralization of hydrogen peroxide [22]. The plasma superoxide dismutase (SOD) activity was estimated by using nitro blue tetrazolium (NBT) as a substrate [23], with certain modifications [24].

### 2.7. Histology of Vital Organs

The samples of vital organs, such as liver, spleen, kidney and intestine (50 mm in length segment from the central part of the small intestines—duodenum and ileum), were collected and fixed in 10% neutral buffered formalin. Two sections from each segment were processed to obtain 4 µm thick paraffin sections, which were stained with haematoxylin and eosin (HE). The slides were evaluated under a Nikon Eclipse Ci-S microscope attached to a camera and the computer software NIS-D Version 4.0 was employed to assess the histological changes.

### 2.8. Statistical Analysis

The data obtained from different parameters were subjected to an independent sample T test using SPSS version 16.0.

## 3. Results

**Figure 2:**
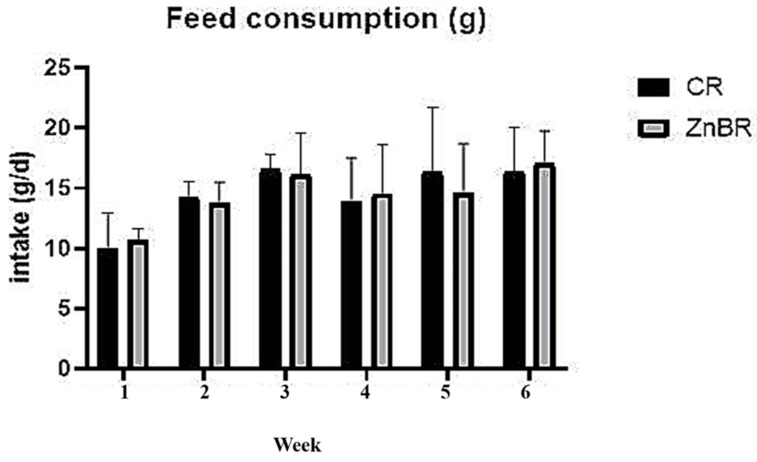
Effects of feeding conventional (CR) and zinc-biofortified (ZnBR) rice-based diets on weekly feed consumption in Wistar rats. Replacing CR with ZnBR did not affect the feed consumption (*p* > 0.05).

**Figure 3:**
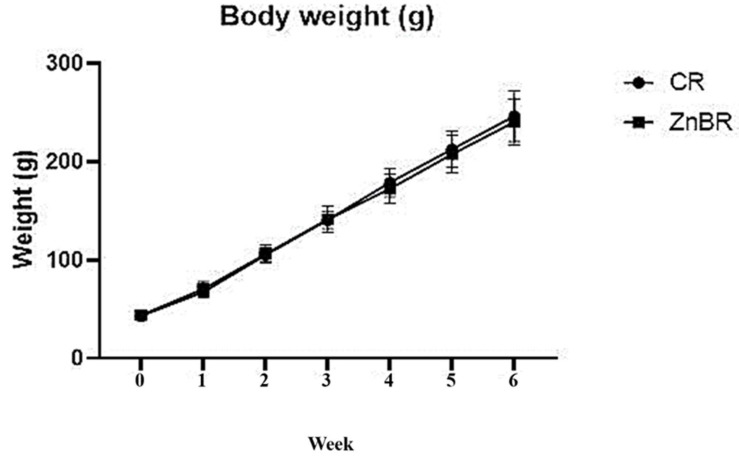
Effects of feeding conventional (CR) and zinc-biofortified (ZnBR) rice-based diets on weekly body weight in Wistar rats. Replacement of CR with ZnBR did not influence the rats’ body weight (*p* > 0.05).

**Figure 4:**
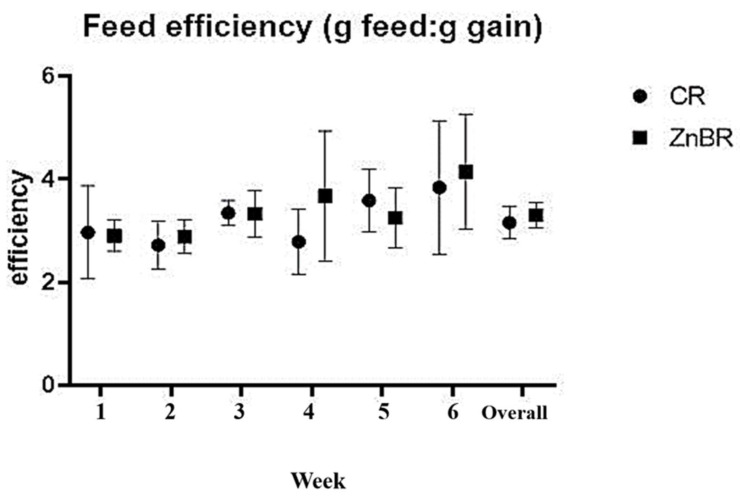
Effects of feeding conventional (CR) and zinc-biofortified (ZnBR) rice-based diets on weekly feed efficiency in Wistar rats. Replacement of CR with ZnBR did not influence the feed efficiency in rats (*p* > 0.05).

**Figure 5:**
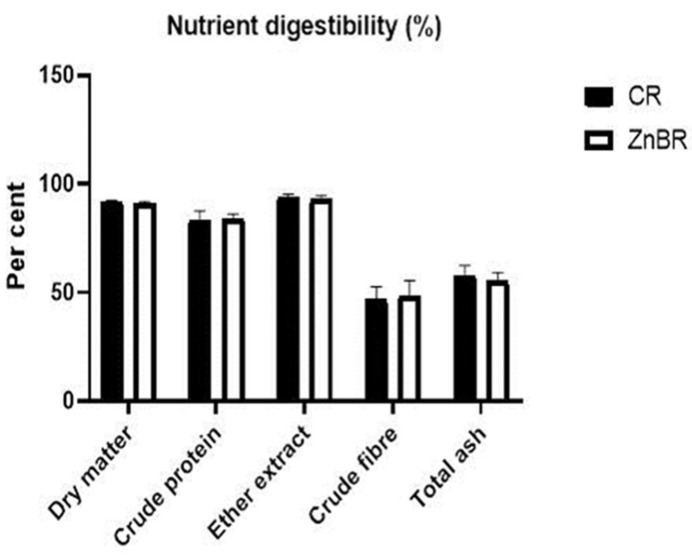
Effects of feeding conventional (CR) and zinc-biofortified (ZnBR) rice-based diets on nutrient digestibility in Wistar rats. Replacement of CR with ZnBR did not affect nutrient digestibility in rats (*p* > 0.05).

**Figure 6:**
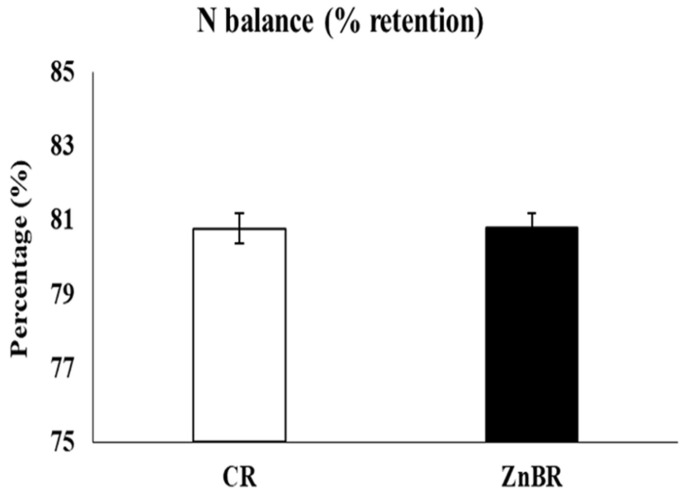
Effects of feeding conventional (CR) and zinc-biofortified (ZnBR) rice-based diets on nitrogen (N) balance in Wistar rats. N balance was comparable among the CR and ZnBR groups (*p* > 0.05).

The experimental diets consisted of 16.16 and 15.68% crude protein and 20.16 and 30.60 ppm zinc in the CR and ZnBR diets, respectively (Table 1). The concentrations of other bivalent cations, Fe and Ca, were similar in the ZnBR and CR (Fe: 6.67 vs. 5.98 ppm; Ca: 0.006 vs. 0.004%). The weekly feed consumption (g/animal/day) was not significantly (*p* > 0.05) different between the CR and ZnBR groups throughout the study periods, indicating acceptance of both rice varieties. Similarly, the initial and final body weights were comparable (*p* > 0.05) between the groups. As well, the consumption and utilization of feed were not significantly different (*p* > 0.05) between the groups (Figure 2, Figure 3 and Figure 4). Further, the feed consumption, body weight and feed utilization efficiency were similar between the male and female rats (Tables S1–S3). The digestion trial revealed comparable digestibility values for DM, CP, EE, CF, and total ash (*p* > 0.05) between the groups (Figure 5). The faecal and urinary N were determined, and the net N retention relative to intake was similar (*p* > 0.05) between the CR and ZnBR groups (Figure 6).

**Figure 7:**
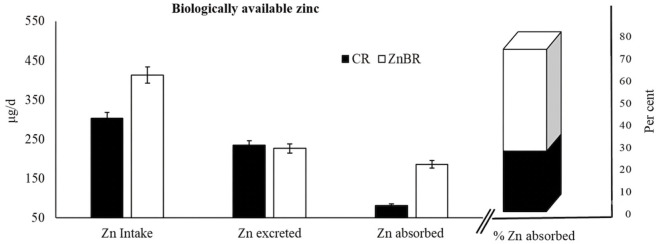
Effects of feeding conventional (CR) and zinc-biofortified (ZnBR) rice-based diets on biologically available zinc (Zn) in Wistar rats. The absorption of Zn was higher in ZnBR-fed rats compared to the CR (*p* < 0.05).

Similar (*p* > 0.05) feed intake (on a DM basis) was observed in both groups, but net Zn intake was higher (*p* < 0.05) in the ZnBR, compared to the CR-fed rats (413 vs. 304 µg/d). With similar (*p* > 0.05) faecal Zn-excretion rates (227 vs. 235 µg/d), the net intestinal absorption of Zn was significantly (*p* < 0.05) higher in ZnBR (45%) than in CR (27%), resulting in 2.3-fold more biologically available Zn in ZnBR-fed rats (Figure 7). The distribution of Zn among various tissues was studied to assess the kinetics of the absorbed mineral (Table 2). The Zn content in various tissues (muscle, liver, plasma, bone and intestine) was similar (*p* > 0.05) in both groups.

Zn plays a vital role in the enzymatic antioxidant defence mechanism in the body; hence, the activities of SOD and CAT were studied (Table 3). The activities of both enzymes were similar (*p* > 0.05), and were not affected by the higher Zn retention in the body.

**Figure 8:**
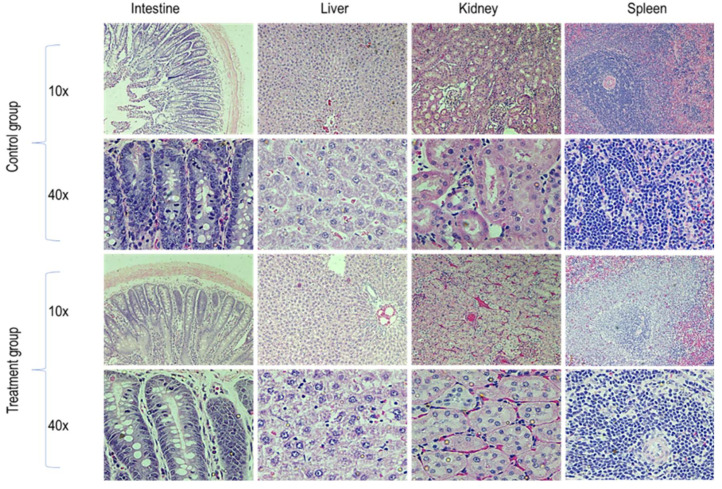
Histological changes in different organs of Wistar rats fed with conventional and zinc-biofortified rice.

The gross morphological examination of vital organs did not indicate any abnormality between the groups. The histological examination of liver tissue revealed no significant microscopic pathological changes, except in one case in the ZnBR group, which showed mild hydrophobic degenerative changes. In contrast, the kidney showed mild congestion in ZnBR compared to the control group (Figure 8). The spleen of the treated group (ZnBR) showed no significant changes in either the white or the red pulp. The jejunum did not show any significant microscopic changes in either experimental group. Further, no deviations/changes in vital organs were observed between male and female Wistar rats.

## 4. Discussion

Biofortification of vital micronutrients (e.g., trace minerals, vitamins, and pigments) is being carried out globally, and specifically in developing countries, through improved genetic selection and the application of advanced molecular tools, to address malnutrition and hidden hunger. Zn is known for its essential role in various physiological processes, and inadequate Zn intake can adversely affect overall bodily functions. Plant breeders have developed biofortified rice and wheat varieties to alleviate this malnutrition, especially in developing countries. India is the world’s largest producer of rice (151 million metric tons per year), and over 70% of its population consumes rice-based preparations as staple food [25]. Biofortification of rice with higher Zn content may ensure adequate mineral intake and minimise malnutrition. The ICAR-Indian Institute of Rice Research has developed a ZnBR rice variety, DRR Dhan 48. Its Zn content is approximately 10 ppm higher than that of the CR variety, which may enhance net Zn intake and help to meet the recommended daily nutrient allowances [26]. However, the concentrations of Fe and Ca remain similar in both the ZnBR and CR varieties [27]. In the current study, complete replacement of CR with ZnBR did not affect feed consumption, body weight change, or plasma Zn content in Wistar rats. The rats in the CR group were fed 20 ppm Zn, whereas growing rats require only 12 ppm Zn [28]. It has been reported that the incorporation of ZnBR in a Zn-deficient rat diet has resulted in a dose-dependent increase (*p* < 0.05) in growth performance [18]. Furthermore, the Zn sourced from ZnBR had a greater impact on growth performance compared to that of from the CR. A linear improvement in growth rate and body weight was observed, as dietary Zn content increased from 01 to 12 ppm. However, further increase above 12 ppm did not yield additional benefits, suggesting that the response to Zn is rate-limiting [29]. Feeding rats excess Zn (24, 1016, 2008 and 3000 ppm) did not affect body weight, feed intake or feed efficiency [30]. As the experimental diet contained a Zn level exceeding 12 ppm, no additional effect on growth performance was observed in the present study.

The Zn content in various tissues (plasma, muscle, bone, liver and intestine) was higher in the ZnBR, compared to the CR group. A previous study indicates that supplementation of Zn through ZnBR significantly increases plasma Zn content in rats fed a Zn-deficient diet, without any change in the total feed intake [18]. These findings suggest that the consumption of ZnBR could alleviate trace mineral malnutrition, and may positively impact growth performance under conditions of malnutrition or Zn deficiency.

The Zn absorption in rats fed the ZnBR diet was 2.3-fold higher (45 vs. 27%) than the CR-fed rats, indicating greater Zn bioavailability from the ZnBR rice. The phytate content in grain negatively affects Zn availability due to the formation of complexes with mineral ligands [31]. The increased Zn availability from intestinal absorption may be attributed to the low phytate-to-Zn molar ratio in the ZnBR [32]. The phytate/phytic acid present in grains can bind to bivalent cations (2^+^), thereby reducing their bioavailability. The non-phytate Zn is more readily available for absorption at the intestinal level than the insoluble phytate-bound Zn. Biofortification of rice resulted in increased Zn content and reduced phytate level (4.93 vs. 7.72 mg/g), as well as a reduced phytate-to-zinc molar ratio (11.97 vs. 31.66) [17]. Previous studies have demonstrated that the concentration of phytate in grains does not directly affect Zn bioavailability. Instead, the phytate-to-Zn molar ratio is the determining factor [32]. The findings of the current study suggest that consumption of ZnBR as a staple food could help in alleviating hidden micronutrient deficiency. The rats are considered to be an established animal model for mineral bioavailability research due to their physiological relevance to humans, especially regarding mineral retention in tissues and blood. However, in humans, Zn bioavailability is mainly influenced by the phytate-to-Zn molar ratio, whereas in rats, it is more closely associated with dietary Zn concentration. In the present study, chemical analysis of biofortified rice revealed a reduced phytate-to-Zn molar ratio, which may have a comparable effect on Zn availability in humans.

The distribution of absorbed Zn among various target organs (muscle, liver and intestine) and plasma was assessed by determining their mineral content. The mineral content was comparable (*p* > 0.05) between the CR and ZnBR groups. Although not significant, Zn content was increased most in plasma (19% ↑), followed by muscle (7.31% ↑) and liver (2.30% ↑), when rats were fed the ZnBR diet compared to the CR diet. In contrast, the bone Zn content was lower (15.6% ↓) in the ZnBR group than in the CR group. Aside from bone, other organs exhibited a marginal increase in Zn content in ZnBR-fed groups, indicating a broader distribution pattern of the more available Zn throughout their bodily systems. Previous studies have indicated that different organs require varying dietary Zn levels to reach plateau concentrations. Plasma Zn content has exhibited a linear increase up to an intake of 31.3 ppm, liver up to 37.1 ppm, kidney up to 28.3 ppm, and femur up to 35.1 ppm. In contrast, the gastrocnemius muscle has not exhibited any trend with different levels of Zn supplementation. Interestingly, the pancreas has exhibited a sharp rise in Zn content, even at a low dietary concentration of 8.40 ppm. Further increase in dietary Zn has resulted in a significant reduction in pancreas weight [30]. The findings of the current study suggest that the higher Zn retention due to the consumption of ZnBR is likely to be distributed among the target organs, potentially leading to an overall increase in an individual’s Zn status. Dietary Zn has a positive impact on immune function in humans [33]. Considering its distribution among major predilection organs, the retained Zn might enhance the development and functioning of the immune system.

Although higher absorption and an increased retention percentage of Zn was observed in the ZnBR-fed group, the increased Zn availability did not influence the activity of mineral-dependent enzymatic antioxidant systems (SOD and CAT). Both the CR- and ZnBR-fed rats received sufficient Zn in their diet, which maintained the basal levels of SOD and CAT. Furthermore, since the rats were maintained under hygienic conditions at an ambient temperature of 24 °C and 50% relative humidity, they did not experience stress, and the activity levels of antioxidant enzymes were similar between the groups. Previous reports indicate that dietary Zn intake at 40 ppm has increased SOD and CAT activity in the serum of Osmanabadi goats [34], and at the tissue level in broiler chickens, at 50 ppm under non-challenged conditions [35]. In rats, supplementation of Zn at 190 or 380 ppm under normal conditions does not affect plasma SOD and CAT activity. However, under induced oxidative stress (chemical or diabetic challenge), Zn supplementation enhanced the activity of both antioxidant enzymes [36]. These findings indicate that Zn intake at the recommended doses or lower does not affect antioxidant enzyme activities under normal conditions, which was also evident in the current study.

Histological examination of vital organs (liver, intestine, spleen and kidney) in the ZnBR group did not reveal any abnormal changes indicative of excess Zn or toxicity. The dose-dependent impact of dietary Zn on histological changes in the liver, kidney and spleen has been studied in mice [37]. A study reported pathological changes in the liver (cell wall loss, lymphocyte infiltration, deformed nuclei, degeneration, and necrosis), kidney (lymphocyte infiltration, constricted or destroyed glomeruli, and structure loss), and spleen (red pulp invading white pulp). Feeding excess Zn (42, 84 and 125 times higher than the basal diet) has not shown an effect on growth, feed intake, feed efficiency, or the weight of vital organs, and no signs of toxicity (vomiting or gastro-intestinal dysfunctions) were evident in rats [30]. The present study did not find any of the above histological changes in Wistar rats. These results suggest that the consumption of ZnBR can improve the body’s Zn status and is safe.

## 5. Conclusions

In conclusion, the biofortified rice had a higher Zn content (8–10 ppm more) compared to the conventional rice variety. Consumption of ZnBR increased the net Zn intake, and higher levels of Zn biological availability were evident in Wistar rats. Nevertheless, the fortified rice-based diet did not improve growth performance, nutrient digestibility, nitrogen balance, mineral content in vital organs or anti-oxidant status, compared to the control. The results indicate that the Zn-biofortified rice varieties could be used to improve marginal deficiency conditions. However, in addition to replacement trials, further studies are needed to correlate Zn content and phytate-to-Zn molar ratio with mineral bioavailability before introducing biofortified rice varieties into the public distribution system to address malnutrition in the human population.

## Figures and Tables

**Table 1:** Chemical composition (%) and zinc content (ppm) in the rice and experimental diets.

Particulars	Rice		Experimental Diet	
	CR	ZnBR	CR	ZnBR
Dry matter (%)	89.21	89.95	95.15	96.46
Crude protein (%)	10.47	9.59	16.16	15.68
Ether extract (%)	2.12	2.17	11.89	11.16
Total ash (%)	0.49	0.73	4.96	4.98
Zinc (ppm)	14.63	24.70	20.16	30.60

CR: Conventional rice (MTU1010); ZnBR: Zinc-biofortified rice (DRR48).

**Table 2:** Effects of zinc-biofortified rice feed on Zn content (ppm) in various tissues.

Group	Muscle	Bone	Liver	Intestine	Plasma
CR	113.7 ± 22.5	223.8 ± 26.7	119.6 ± 14.7	258.3 ± 59.2	1.11 ± 0.26
ZnBR	122.0 ± 34.9 (+7.31%)	188.8 ± 10.7 (−23.85%)	122.3 ± 16.0 (+2.30%)	198.8 ± 35.1 (−29.84%)	1.32 ± 0.13 (+18.92%)
*p* value	0.845	0.251	0.902	0.407	0.480

The values in parentheses indicate the percent change in mineral content in the biofortified group over the conventional diet. CR: Conventional rice (MTU1010); ZnBR: Zinc-biofortified rice (DRR48).

**Table 3:** Effects of zinc-biofortified rice feed on the activity of plasma antioxidant enzymes (U/mL) in Wistar rats.

Antioxidant Enzymes	CR	ZnBR	*p* Value
Superoxide dismutase	23.6 ± 0.53	24.0 ± 0.49	0.457
Catalase	0.11 ± 0.02	0.12 ± 0.01	0.381

CR: Conventional rice (MTU1010); ZnBR: Zinc-biofortified rice (DRR48).

## Data Availability

The original contributions presented in this study are included in the article/Supplementary Materials. Further inquiries can be directed to the corresponding authors.

## Supplementary Materials

The following supporting information can be downloaded at https://www.mdpi.com/article/10.3390/foods15142567/s1, Table S1: Effects of feeding conventional (CR) and zinc-biofortified (ZnBR) rice-based diets on feed consumption (g/d/rat) in Wistar rats (sex wise). Table S2: Effects of feeding conventional (CR) and zinc-biofortified (ZnBR) rice-based diets on body weight in Wistar rats (sex wise). Table S3: Effects of feeding conventional (CR) and zinc-biofortified (ZnBR) rice-based diets on feed efficiency (g feed:g gain) in Wistar rats (sex wise).
