# Supplementary material for: Consumption of Zinc Biofortified Rice Diet Improved Mineral Availability: An Approach to Address Micronutrient Malnutrition
Source: Foods. 2026 Jul 22;15(14):2567. doi: 10.3390/foods15142567 (PMC13407753; doi:10.3390/foods15142567)
Supplement: Supplementary file 1 [file foods-15-02567-s001.zip › foods-4267030-supplementary.pdf]

Supplementary data

Table S1: Effects of feeding conventional (CR) and zinc-biofortified (ZnBR) rice-based diets on feed consumption (g/d/rat) in Wistar rats (sex wise).

Age	Feed consumption					
	Male		P value	Female		P value
	Control	Biofortified		Control	Biofortified	
Day 7	10.9±0.49	15.7±1.05	0.851	9.03±1.18	12.2±0.38	0.212
Day 14	10.8±0.39	16.8±1.03	0.532	10.7±0.21	12.3±0.40	0.310
Day 21	14.7±0.35	19.6±0.97	0.882	13.9±0.39	13.1±0.50	0.110
Day 28	14.2±0.63	17.0±0.81	0.503	13.2±0.45	12.2±0.54	0.807
Day 35	17.3±0.17	18.7±0.43	0.071	15.8±0.29	14.1±0.54	0.281
Day 42	17.5±1.25	18.6±0.52	0.929	14.9±0.42	15.5±0.44	0.075

Table S2: Effects of feeding conventional (CR) and zinc-biofortified (ZnBR) rice-based diets on body weight in Wistar rats (sex wise).

Age	Body weight (g)					
	Male		P value	Female		P value
	Control	Biofortified		Control	Biofortified	
Day 0	43.8±1.58	43.0±1.27	0.709	42.9±1.87	42.3±1.42	0.782
Day 7	68.5±2.87	68.4±2.88	0.983	65.7±2.87	68.7±2.78	0.461
Day 14	109±4.13	105±3.95	0.436	99.2±3.18	99.4±3.78	0.968
Day 21	146±5.19	140±6.08	0.440	131.6±3.44	132±4.39	0.868
Day 28	190±6.86	174±6.92	0.115	160±3.71	156±4.93	0.526
Day 35	227±7.61	214±8.36	0.258	187±5.30	181±5.49	0.402
Day 42	268±9.23	252±9.22	0.229	210±5.55	203±5.87	0.418

Table S3: Effects of feeding conventional (CR) and zinc-biofortified (ZnBR) rice-based diets on feed efficiency (g feed : g gain) in Wistar rats (sex wise).

Age	Feed efficiency					
	Male		P value	Female		P value
	Control	Biofortified		Control	Biofortified	
Day 7	3.25±0.08	3.29±0.28	0.883	3.04±0.53	3.11±0.20	0.916
Day 14	2.54^{b}±0.08	2.75^{a}±0.04	0.041	2.84±0.08	3.06±0.12	0.153
Day 21	3.30±0.09	3.58±0.15	0.154	3.43±0.08	3.17±0.11	0.083
Day 28	2.58±0.21	3.72±0.59	0.106	3.00^{b}±0.17	3.74^{a}±0.06	0.003
Day 35	3.74^{a}±0.12	3.03^{b}±0.15	0.006	3.35±0.20	3.53±0.15	0.483
Day 42	3.36±0.30	3.50±0.07	0.662	4.48±0.44	4.90±0.27	0.437
